# Everybody's Page

**Published:** 1890-02-22

**Authors:** 


### Everybody's Page.

Out of 8,000 cabmen in the metropolis only 1,200 subscribe to the Cab-drivers' Benevolent Association.

---

It has been observed that the influenza epidemic in Sweden seemed more to follow the channels of communication than the atmospheric currents. Beginning in Stockholm, it followed the railway lines to the larger towns, afterwards spreading to smaller towns and country places, and last of all reaching the isolated and remote Norrland.

---

It has come to light recently in America that dirt under the name of "Terra Alba" is used to an enormous extent in the manufacture of candy. Terra alba is a mineral which is absolutely insoluble in either the gastric juice or the saliva, and when we learn that 6,000 tons of terra alba were just lately imported into New York alone our feelings cause us to exclaim, "Poor little American children with a liking for candy!"

---

The daily average of visitors to the People's Palace is about 1,100, and at present the number of books for their use does not amount to 12,000, although the officials say they would like 250,000. The Clerkenwell Free Library's return as to what books are most in request shows that out of 47,000 volumes issued to readers over 37,000 volumes were novels. Biography and history come next, but a long way behind, numbering only 2,516. Poetry does not seem to appeal to the readers at all.

---

Mr. John Sinclair, writing from the Working Men's College, Great Ormond-street, to the *Times*, states that on March 5th Lord Brassey will preside at a conference at the Memorial Hall, Farringdon-street, to consider of the improvement and further provision of open spaces devoted to games in and near London. We wish the conference much success in rescuing open spaces from the fate of becoming covered with "desirable villas," and in obtaining grounds where lads and men who have been cooped up all the week in stuffy City offices can enjoy their Saturday afternoon football or cricket.

---

Beware of Bologna sausages! The alleged discovery that "some amongst the largest manufacturers of Bologna sausages have been in the habit of using horseflesh, not of the first quality, mixed with inferior pork, in the manufacture of sausages" is certainly calculated to "cause considerable excitement among the population"; we are only surprised that this doubtless tasty but inferior article of food has not caused considerable illness also. Then there was the case last week of the omnibus driver who was charged with driving animals unfit for work. The auctioneer stated to the magistrate that he had not sold the horses for work, but to be shipped abroad to be converted into beef-tea and sausages!

---

Writing lately to a Band of Hope in Scotland Miss Florence Nightingale said: "Don't think you can do anything worth doing in a fit of enthusiasm," and this enthusiasm, which, unfortunately, is very momentary, is met with very much too often. It is too much the fashion to dabble in things, no matter what, from philanthropy to playing on the banjo. People *rush* at things pell-mell, and enjoy them thoroughly, or think they do, for a few weeks, and then away goes "the fit" as fast as it came, which when it's only a question of banjo playing doesn't matter. But it's a pity to promise help to any cause worth helping, then find out after a bit that it takes too much time, which being interpreted means "it isn't quite as interesting as my enthusiasm led me to believe it would be."

---

As long as three hundred years ago a Spanish traveller described the use of coca. The Indians chew a paste made of the leaves of the plant in order to be able to endure fatigue, and to keep off hunger and thirst whilst they are on their long expeditions.

---

Dr. Arapad Bokai is said to have compounded a solution which completely neutralizes the poison brought into the system by the bite of a mad dog. We are all very much obliged to Doctor Bokai, and should like to hear news of any people who have benefited by the treatment. The solution is composed of chlorine water, salt brine, sulphurous acid, permanganate of potassium, and eucalyptus oil.

---

Dr. Fisk, of Denver, has noticed that the number of men who secure arrest of pulmonary disease by going to Colorado is far greater than that of women. He attributes this in great measure to the fact that a man bears separation from home and difference of surroundings more easily than a woman. This seems a good reason, for there is nothing a woman feels harder to bear than illness away from her home.

---

There is an ancient proverb which says: "When you plant the olive you pray it to give you fruit; when you manure it you not only pray, but you entreat; but when you prune it you compel it to give the fruit." But unfortunately the truth of this proverb is sometimes undone by a wretched little parasite called the "dacus," the larva of which is hatched out in the fruit. It is nourished by the substance of the latter, and frequently causes destruction on a large scale.

---

Before the speakers came on to the platform at the meeting of the Women's Trades Association, we overheard one of the audience, a tailoress, discussing Board schools with those in the seats near, and she said she disapproved strongly of the children being taught Scotch in the Board schools, which remark rather electrified some of the listeners. She was assured she was mistaken, but no! the good lady knew best, and declined to believe anybody. We could only imagine she had heard the children sing "Bonnie Dundee," or "Auld Lang Syne."

---

The meeting of the Academie de Medicine of the 28th adopted M. Bergeron's conclusions on the contagion of Tuberculosis. The greatest danger appears to be in the fact that the dust which remains when the expectoration of a tuberculous person has dried contains an infinite quantity of germs. Dr. Cornet, of Berlin, who is of the same opinion, insisted at the Strasburg Congress how important it was that phthisical people should not expectorate on to the ground nor into their handkerchiefs, but into a vessel containing water; and that these ought to be in all public places, and that the present system of using sawdust and sand in dry receptacles is much to be condemned. Sanitarians would do well to bear this in mind.

---

Mr. Robinson's book, "Cremation and Urn-Burial," is full of strong argument, and in some senses, persuasive argument. "The Cemetery of the Future" which he describes would be as lovely, indeed, far lovelier, than any of our existing cemeteries or graveyards; for inasmuch as urn-burials would not occupy more than a fourth of the space of a large cemetery, all the rest would be for gardens and groves of trees. There is something repulsive in the knowledge that the remains of those we have loved will in time become a source of danger to the living. "By the complete rapid progress of cremation," urges Mr. Robinson, "we ensure the purity of the air and water for the benefit of the living."

